# Actin dynamics controlled by IqgC, a RasGAP at the crossroads between the IQGAP and fungal GAP1 families

**DOI:** 10.1002/2211-5463.70083

**Published:** 2025-07-11

**Authors:** Vedrana Filić, Darija Putar, Lucija Mijanović, Igor Weber

**Affiliations:** ^1^ Division of Molecular Biology Ruđer Bošković Institute Zagreb Croatia; ^2^ Division of Biosciences University College London UK

**Keywords:** cell adhesion, cell migration, *Dictyostelium*, macropinocytosis, Rap, Ras

## Abstract

In addition to transmitting receptor‐mediated signals to adjust the gene expression profile of the cell, small GTPases of the Ras family also control the remodelling of the actin cytoskeleton. The conversion of Ras GTPases from their active to their inactive form is controlled by Ras GTPase‐activating proteins (RasGAPs). IqgC, a RasGAP from *Dictyostelium discoideum*, was originally assigned to the IQGAP family, but its sequence and recent functional analyses show that IqgC is more closely related to RasGAPs from the GAP1 family of fungi. IqgC has two prominent domains, a RasGAP domain and a C‐terminal RGCt domain, and interacts with Ras, Rab and Rap GTPases, but shows GTPase‐promoting activity only towards Ras. IqgC suppresses macroendocytosis but supports cell‐substratum adhesion and directed cell migration. Its localisation to macroendocytic cups is mediated by the RasGAP domain, whereas its localisation in ventral focal adhesions is mediated by the RGCt domain. We hypothesise that IqgC plays an important role in the balance between the competing feeding and migratory behaviour of amoeboid *D. discoideum* cells.

## Graphical abstract

IqgC is a RasGAP from *Dictyostelium discoideum*. IqgC binds RasG via its RasGAP domain and deactivates it on macroendocytic cups, thereby suppressing the uptake of fluid and particles. IqgC has a positive effect on cell‐substratum adhesion, and its RGCt domain is required for recruitment to ventral foci. IqgC also interacts with RapA, and both GTPases have a stimulatory effect on adhesion.

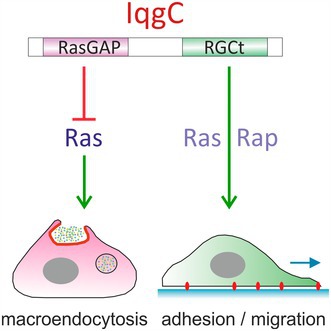

**Abbreviations**
BiFC: bimolecular fluorescence complementation
CAF: cancer‐associated fibroblasts
CH: calponin homology
CT domain: C‐terminal domain
fMLP: *N*‐formyl‐methyl‐leucyl‐phenylalanine
GRD: GAP‐related domain
IQGAP: IQ motif‐containing GTPase‐activating protein
NAF: normal associated fibroblasts
NF1: neurofibromin 1
Nox2: NADPH oxidase 2
PH: pleckstrin homology
PI(3,4,5)P_3_
: phosphatidylinositol (3,4,5)‐trisphosphate
PI3K: phosphoinositide 3‐kinase
RasGAP: Ras GTPase‐activating protein
RGCt: RasGAP C‐terminal

## RasGAPs vs. IQGAPs—similarities and differences

Ras GTPase‐activating proteins (RasGAPs) are negative regulators of small, monomeric GTPases of the Ras family. Ras GTPases are often referred to as molecular switches because they toggle between the GTP‐bound ‘on’ state, which can transmit signals, and the GDP‐bound ‘off’ state, which is inactive [1]. The transition from the active to the inactive state depends on GTP hydrolysis, which is immensely facilitated by RasGAPs. RasGAPs are modular proteins of varying sizes that share a common RasGAP catalytic domain, usually surrounded by C2, pleckstrin homology (PH), calponin homology (CH) and Sec14‐like domains, mostly involved in their regulation and membrane translocation [2]. The RasGAP domain catalyses GTP hydrolysis at the active site of Ras by stabilising the transition state [3]. Upon binding of Ras to its GAP, the conserved catalytic arginine of GAP, termed the arginine finger, inserts into the phosphate binding site and stabilises the transition state of the phosphoryl transfer reaction by neutralising the negative charge of the phosphate. The reaction is further stabilised by the interaction with the secondary arginine/lysine residue of GAP and additional residues that support the conformation of Ras that is favourable for GTP hydrolysis.

The human genome harbours 14 RasGAP genes, whose protein products are divided into six groups: (a) p120GAP/RASA1, (b) neurofibromin, (c) GAP1, and (d) SynGAP family members, (e) plexins and (f) IQGAPs [3]. Members of the GAP1 and SynGAP families, and probably plexins, have dual GAP activity towards Ras and Rap GTPases [4, 5, 6], whereas the RasGAP domains of IQGAP family members are not catalytically active [7, 8, 9]. Nevertheless, the name IQGAP, an abbreviation for IQ motif‐containing GTPase‐activating protein, remained in use, although IQGAPs are not true RasGAPs. They probably lost their catalytic activity early in evolution in an opisthokont ancestor from which fungi and animals branched off [10]. Comparison of the structures of the GAP‐related domain (GRD) of human IQGAP1 and the RasGAP domain of human RASA1 has revealed mutations responsible for the loss of GAP activity in the IQGAP family [11, 12]. Instead of the arginine finger in RasGAPs, IQGAPs have a conserved threonine residue (threonine‐1046 in human IQGAP1). In addition, the conserved FLR motif (FLRXXXPAXXXP, from phenylalanine‐901 in human RASA1), which contains residues important for binding to Ras, is converted to a YYR motif (YYRXXXPAXXXP, from tyrosine‐1192 in human IQGAP1) in IQGAPs. Therefore, IQGAPs not only lack RasGAP activity, but also do not bind Ras GTPases via their GRD.

IQGAPs are multidomain proteins containing the N‐terminal CH domain and the coiled coil (CC) repeat region, centrally located WW and IQ domains, GRD and RasGAP C‐terminal (RGCt) domains in the C‐terminal half of the protein and an extreme C‐terminal (CT) domain [13, 14]. IQGAPs bind a large number of partners via these domains; for example, human IQGAP1 has more than 150 identified interactors [15]. These include the GTPases Cdc42 and Rac1, which make IQGAPs effectors of the Rho family GTPases [16]. Early studies have shown that the GRD is necessary but not sufficient for high‐affinity binding to Cdc42 and Rac1 [8, 17, 18]. In a more recent study, a multiple‐step binding mechanism involving sequential binding to RGCt, GRD and CT has been proposed [14, 19]. Since IQGAPs interact with numerous binding partners, they act as molecular scaffolds [15]. Initially, they were perceived as modulators of the actin cytoskeleton [20], but later studies showed that IQGAP1 binds components of various signalling pathways and thus modulates their activity. Therefore, IQGAPs regulate various cellular processes such as migration [21, 22, 23, 24], adhesion [23, 25, 26], cytokinesis [27], phagocytosis [24, 28] and macropinocytosis [29] and are important for the physiology of the whole organism [30].

*Dictyostelium discoideum* is an amoebozoon that serves as a model organism for a number of biological processes, including cell motility and chemotaxis, actin cytoskeleton dynamics and associated signalling mechanisms [31]. *D. discoideum* is genetically tractable, its genome has been sequenced, and many functional assays are well‐established [32]. The genome of *D. discoideum* encodes at least 18 RasGAP domain‐containing proteins [33, 34], including IqgD (UniProtKB AC: Q552W4). Based on the GRD‐RGCt domain organisation characteristic of the IQGAP protein family [10], four RasGAP domain‐containing proteins, DGAP1, GAPA, IqgC and IqgD, have been classified as the *D. discoideum* IQGAP family [35]. Except for IqgD, the other three proteins lack the N‐terminal domains and are much shorter than IQGAPs from higher eukaryotes. Therefore, they are often referred to as IQGAP‐related proteins. However, a more detailed analysis of the amino acid sequence of IqgC, followed by experimental evidence, revealed that, unlike DGAP1 and GAPA, IqgC has conserved RasGAP catalytic activity that is unique among nominal IQGAPs [36].

We have shown that IqgC binds three small GTPases of the Ras superfamily: RasG, RapA and Rab5 [36, 37, 38]. While RasG is a specific target for its GAP activity, IqgC is not a GAP for the other two. Here, we review the biological role of IqgC in macroendocytosis, adhesion and motility and revise its position within the IQGAP family.

## IqgC negatively regulates macroendocytosis via small GTPase RasG

**Fig. 1:**
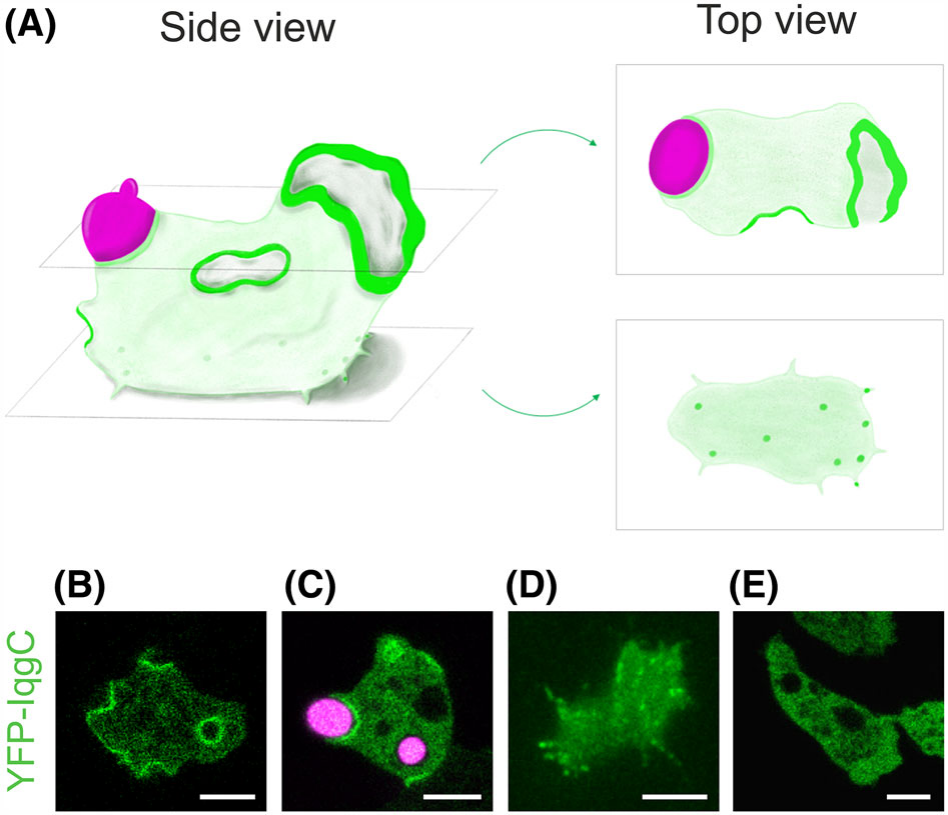
IqgC localises to macroendocytic cups, ventral adhesion foci and hyaline zones in leading pseudopodia in vegetative *D. discoideum* cells. (A) Side view of a schematic representation of a vegetative cell expressing fluorescently labelled IqgC (green) taking up a TRITC‐labelled yeast cell (magenta) (*left*), and top view of two horizontal cross sections (*right*): One near the dorsal cell surface (*top right*) and the other near a substrate (*bottom right*). (B) Confocal sections showing the localisation of fluorescently labelled IqgC (green) in macropinocytic cups, (C) phagocytic cups, (D) ventral adhesion foci and (E) actin‐rich hyaline zones in leading pseudopodia of a randomly moving vegetative *D. discoideum* cell. Scale bars in panels B–E, 5 μm.

In vegetative *D. discoideum* cells in the growth phase, fluorescently labelled IqgC is localised to macropinocytic cups and nascent macropinosomes and, to a lesser extent, to phagocytic cups and phagosomes (Fig. 1A–C). This localisation and the presence of the conserved arginine finger (arginine‐205) and the almost completely conserved FLR motif (LLRYINPAVVTP from leucine‐351) suggested that IqgC could be a RasGAP that acts as a negative regulator of Ras activity during macroendocytosis. Indeed, the yeast two‐hybrid assay showed a specific interaction with constitutively active RasG [36]. Furthermore, IqgC does not interact with Rho family GTPases, in contrast to DGAP1, GAPA and other IQGAPs. The interaction of IqgC with RasG was confirmed in biochemical assays and in living *D. discoideum* cells using the bimolecular fluorescence complementation (BiFC) approach. Its RasGAP activity against RasG was demonstrated in an *in vitro* GAP assay with purified proteins. Depletion and overexpression of IqgC in wild‐type cells showed that the amount of IqgC in the cell correlated negatively with macropinosome size and macropinocytosis efficiency, resulting in better growth of the knockout mutant in liquid culture medium [36, 37]. We have also shown that IqgC binds to RasG via its RasGAP domain and that this interaction is crucial for the localisation of IqgC to macropinocytic and phagocytic cups [37]. These results confirmed that IqgC interacts specifically with RasG during macropinocytosis. Mutation of the arginine finger or the FLR motif in IqgC prevented its interaction with RasG and its correct localisation.

The amount of IqgC in cells also correlated negatively with the efficiency of phagocytosis. In particular, the uptake of large particles was significantly increased in the absence of IqgC, whereas the effect was less pronounced during the clearance of bacteria from suspension [36]. However, the stronger effect on phagocytosis than on macropinocytosis is surprising because recruitment of IqgC to phagocytic cups is weaker than its recruitment to macropinocytic cups. A possible explanation could be that the influence of IqgC on phagocytosis is independent of its interaction with RasG on phagosomes. As mentioned above, IqgC also binds RapA, which is known to increase phagocytosis and decrease macropinocytosis [39]. However, unlike RasG, this binding is nucleotide‐independent and is mediated by both the RasGAP and RGCt domains of IqgC, reminiscent of IQGAP1‐Cdc42 binding [38]. It is therefore possible that IqgC negatively regulates phagocytosis by sequestering RapA.

A comparison of IqgC with three other *D. discoideum* RasGAPs involved in macroendocytosis, NF1, RGBARG and C2GAP2, has been presented recently [37]. Here we only mention the recently identified *D. discoideum* complex Leep2, a heterodimer consisting of Leep2A and Leep2B [34]. These two proteins show sequence homology to human RalGAPA1/RalGAPA2 and RalGAPB, respectively, which together form two RalGAP complexes that function as GAPs for the human Ras‐like GTPases RALA and RALB [40, 41]. However, in *D. discoideum*, Leep2 acts as a RasGAP for RasB, RasG and RasD, with the highest GAP activity towards RasG. Deletion of the complex reduces the frequency and size of macropinosomes and thus negatively regulates fluid uptake by macropinocytosis. However, overexpression of Leep2 also reduces the efficiency of macropinocytosis. Leep2 localises to macropinocytic cups and nascent macropinosomes. Interestingly, similar to IqgC, Leep2 is not localised at the leading edge and pseudopodia in randomly moving cells, but is cytosolic with enrichment in the actin‐rich hyaline zone. It is also faintly visible on phagocytic cups.

A correlation between increased Ras activity and membrane ruffling coupled to macropinocytosis was demonstrated in mammalian cells almost four decades ago [42]. Today, we know that actin polymerisation around signalling patches containing active Ras and PI(3,4,5)P_3_ drives macropinosome formation [43]. Nevertheless, our knowledge about the involvement of RasGAPs in the regulation of macropinocytosis in mammals is still quite limited. In fact, a pivotal role of RasGAPs in macropinocytosis was first demonstrated in *D. discoideum* for RasGAP NF1, an orthologue of human neurofibromin 1 [44]. By suppressing the activity of several Ras GTPases, NF1 limits the size of both macropinocytic and phagocytic cups [44, 45]. To date, a negative role in the regulation of macropinocytosis in mammalian cells has only been demonstrated for neurofibromin 1 and RASAL3 [46, 47, 48]. Neurofibromin 1 (NF1) is the most extensively studied RasGAP in mammals. It is ubiquitously expressed, with the highest expression in the nervous system. Germline mutations in *NF1* are associated with the complex multisystem disorder neurofibromatosis type I [49]. In a recent study, bone marrow‐derived macrophages from *Nf1* knockout mice were shown to exhibit significantly increased membrane ruffling and macropinocytosis [47]. Increased Ras/PI3K signalling in the absence of Nf1 leads to increased activation of protein kinase C, which together with active Rac1 promotes assembly of NADPH oxidase 2 (Nox2). The assembled Nox2 complex generates reactive oxygen species that promote cofilin activation, dendritic actin polymerisation, membrane ruffling and macropinocytosis [50, 51]. RASAL3, a member of the SynGAP family that is predominantly expressed in bone marrow and lymphoid tissue, is another example of a RasGAP whose downregulation increases macropinocytosis [48, 52]. The expression of RASAL3 is significantly suppressed in human prostatic cancer‐associated fibroblasts (CAF) compared to normal associated fibroblasts (NAF). Prostatic CAF and prostatic fibroblasts derived from *Rasal3* knockout mice have increased Ras activity and increased macropinocytosis, whereas both NAF and control mouse fibroblasts do not exhibit macropinocytosis.

Similar to macropinocytosis, data on the role of mammalian RasGAPs in the regulation of phagocytosis are quite limited. However, in contrast to macropinocytosis, phagocytosis appears to be downregulated by depletion of RasGAPs [53, 54, 55]. Studies using microglia from heterozygous *Nf1* mutant mice or bone marrow‐derived macrophages from mice carrying the *Nf1*‐Q181X nonsense mutation both showed reduced phagocytosis compared to wild‐type controls [53, 54]. Similarly, macrophages isolated from *Rasa4* knockout mice showed impaired actin polymerisation and phagocytic cup formation [55]. CAPRI/RASA4 is a member of the Gap1 RasGAP family with a broad tissue distribution [56]. In wild‐type mouse macrophages, Rasa4 localises to the phagocytic cups during FcγR‐mediated phagocytosis. However, the effect of RASA4 on phagosome formation appears to be mediated by its interaction with Rho family GTPases, since its RasGAP domain also interacts with Cdc42 and Rac1 in a nucleotide‐independent manner. This interaction is important for their recruitment to the cup and the regulation of actin polymerisation that drives cup formation. Consistently, overexpression of active Cdc42 or Rac1 enhances phagocytosis in both wild‐type and *Rasa4*‐deficient macrophages [55].

## IqgC positively regulates adhesion and directed migration

The local interaction of *D. discoideum* cells with the underlying substratum is mediated by punctate ventral structures that resemble integrin‐based focal adhesions in metazoan cells [57]. Although different transmembrane receptors take over the role of integrins in *D. discoideum*, many other components of the focal adhesion complexes are conserved, for example paxillin, talin and vinculin. Compared to the integrin‐based focal adhesions, the ventral adhesion foci in *D. discoideum* are more volatile and have a lifespan in the order of 1–2 min. We have recently shown that the attachment of *iqgC* knockout cells to hydrophobic, hydrophilic and positively charged surfaces is impaired, suggesting that IqgC is involved in innate regulatory mechanisms of cell adhesion [38, 58]. We also found that IqgC is recruited to ventral adhesion foci (Fig. 1A,D). Interestingly, the RGCt domain is sufficient for the incorporation of IqgC into ventral foci, which is possibly mediated by RapA, but the interaction of the RasGAP domain with RasG appears to positively regulate IqgC turnover. The presence of the fully functional RasGAP domain is also required to support the attachment of cells exposed to shear stress.

IqgC is recruited to the ventral adhesion foci approximately 4 s after paxillin B [38]. Two other components of the focal adhesion complex in *D. discoideum*, talin A and myosin VII, have been identified in the IqgC interactome, and it is possible that they mediate the proximal interactions during IqgC recruitment. However, paxillin B appears to be critical for the stable incorporation of IqgC, as IqgC was not detected in the ventral foci of *paxB* knockout cells. IqgC likely contributes to the stability of the ventral adhesion foci, as the elimination of IqgC led to a shortening of their lifespan. The elimination of talin A or myosin VII resulted in similar premature disassembly of the foci, suggesting that each of the three proteins contributes to the stability of the adhesion complex to a similar extent. Overall, our results suggest that IqgC is an important regulator of the assembly, stability, and disassembly of ventral complexes that control cell–substratum adhesion in *D. discoideum*.

Genetic elimination of IqgC leads to a slight increase in cell velocity during random migration and chemotaxis to cAMP [38]. More importantly, the directed persistence of random migration is drastically reduced in the mutant cells. Given its role in supporting the stability of ventral adhesion foci, it is possible that IqgC also regulates the spatially coordinated formation and removal of these foci, thereby influencing the persistence of cell locomotion. Indeed, it is well‐documented that stabilisation of newly formed protrusions by attachment to the substratum facilitates directed migration [59, 60, 61].

It is currently not clear to what extent IqgC affects cell migration through its influence on cell‐substratum adhesion and to what extent it modifies Ras‐mediated actin polymerisation during leading edge protrusion. It is known that Ras activity is required for the formation of cortical protrusions during both migration and macroendocytosis [62, 63]. It has therefore been suggested that macropinocytosis and migration are largely incompatible [64]. More specifically, a low local concentration of active Ras leads to the formation of pseudopodia, whereas a high concentration promotes the induction of macropinocytic cups [65]. Our results support this notion, as the level of exogenously expressed IqgC correlates positively with cell speed [38], whereas it correlates negatively with the efficiency of macropinocytosis [36]. Also, whereas IqgC is strongly localised on macropinocytotic cups (Fig. 1B), only its weak accumulation in the hyaline, actin‐rich zones in leading pseudopodia has been observed (Fig. 1E) [36]. We therefore hypothesise that the coordination between global and local IqgC‐mediated deactivation of RasG influences the balance between migration and macropinocytosis. Consistently, it has recently been shown that the effects of Ras suppression by rapid recruitment of RasGAPs C2GAPB in *D. discoideum* and RASAL3 in neutrophils and macrophages at the cell membrane differ depending on whether recruitment is local or global [66].

Although IQGAPs have been identified as components of integrin adhesion complexes in mammalian cells, their possible structural or regulatory role remains largely unexplored [25, 26]. It is interesting to note, however, that the interaction between IQGAP1 and Hax1 mediated by the RGCt domain plays a significant role in focal adhesion turnover and directed migration in MCF7 cells [23]. Of the mammalian RasGAPs, RASA1 has been detected as a component of the integrin‐associated complex in fibroblasts and haematopoietic cells [67] and has been shown to localise to the adhesion sites of hepatocellular carcinoma cells [68]. It has been suggested that RASA1 is important for cell polarisation and motility by regulating the realignment of focal adhesion independently of its role in Ras regulation [69]. These effects are mediated by the SH2 and SH3 domains at the N terminus of RASA1. An unexpected role of RASA1 in the recycling of endocytosed integrins to the plasma membrane has been demonstrated in breast cancer and murine endothelial cells [70, 71]. Overall, the role of IQGAPs and RASA1 in cell adhesion and migration in mammalian cells does not appear to be functionally related to the role of IqgC in these processes.

The members of the mammalian GAP1 family (not to be confused with the RasGAP family of the same name in fungi), GAP1^m^/RASA2 and GAP1^IP4BP^/RASA3, have been identified as important regulators of T cell activation and adhesion by regulating the activities of Ras and Rap1 [72]. In endothelial cells, depletion of the dual Ras/RapGAP RASA3 increased the size and lifespan of focal adhesions due to impaired dynamics of their assembly and disassembly as a result of Rap1 hyperactivation [73]. While the effect of RASA3 depletion on the focal adhesion dynamics is opposite to the effect of IqgC depletion in *D. discoideum*, the functional consequences of its interaction with RapA are currently unclear [38]. Another member of the mammalian GAP1 family, CAPRI/RASA4, has been shown to be important for chemoattractant‐triggered, nonadaptive Ras activation in human neutrophils [74]. In *rasa4* knockdown cells, Rap1 was hyperactive and its activity was prolonged upon fMLP stimulation, along with increased adhesion [74]. Also, the calcium‐dependent recruitment of RASA4 to the plasma membrane decreased LFA‐1 integrin–mediated T cell adhesion by inhibiting Rap1 activity [75]. In both cases, the membrane localisation of RASA4 depended on the presence of the C2 and PH domains and not on the RasGAP domain.

## IqgC is closely related to the GAP1 family of RasGAPs from fungi

IqgC was originally assigned to the IQGAP family of *D. discoideum*, and its renaming to DdIQGAP3 has even been proposed [76]. However, a comprehensive phylogenetic analysis of proteins possessing a RasGAP domain has shown that IqgC is more closely related to a group of RasGAPs from fungi, the GAP1 family [10]. The confusion likely arose because both IQGAPs and GAP1s share a RasGAP‐RGCt domain architecture that is not present in any other group of RasGAPs, suggesting that they likely descended from a common ancestor [10]. The GAP1 family of fungi has escaped the attention of researchers in the field of Ras signalling and has not even been mentioned in recent reviews on RasGAPs [3, 72, 77, 78]. The family was named after the founding member from the fission yeast *Schizosaccharomyces pombe* [79, 80], and since then GAP1 orthologues have been identified and characterised in about a dozen species of filamentous fungi [81, 82, 83, 84, 85, 86]. GAP1 family members are involved in the regulation of polarised growth and infection‐associated morphogenesis in various fungal pathogens [87, 88, 89].

Mechanistically, GAP1s play an important role in the deactivation of Ras in fungi, and the GAP1‐deficient strains phenotypically resemble the mutants with constitutively active Ras [85, 90]. During polarised hyphal growth in filamentous fungi, Ras activity controls Cdc42‐mediated actin polymerisation [91, 92]. The deepest insights into the role of a GAP1 were gained in studies of pheromone‐induced shmoo growth during sexual mating in *S. pombe* [93, 94]. During this process, several zones of Ras activity appear and disappear along the cell cortex until one zone stabilises and triggers the growth of a shmoo protrusion towards a partner cell, which undergoes an analogous transfiguration, eventually leading to cell fusion [95]. The formation of these patches is initiated by the local autocatalytic production of Ras‐GTP. Subsequently, GAP1 binds to the membrane‐bound Ras‐GTP and deactivates it. The competition between these two processes ultimately determines whether the patch of active Ras disappears or triggers a shmoo. A reaction–diffusion model predicts that the extracellular pheromone concentration and the relative diffusion rate between GAP1 and Ras‐GTP play the decisive role in this process [96]. A patch of activated Ras is only stabilised when the level of the pheromone signal is increased by proximity to a neighbouring cell, leading to the activation of downstream signalling mechanisms that trigger the growth of a shmoo. Interestingly, IqgC is also involved in controlling the dynamics of spatially delineated cytoskeletal regions in the cortex of *D. discoideum* cells, namely the macroendocytic cups and the ventral focal patches [36, 38]. It remains to be investigated whether these processes have a common mechanistic basis in *S. pombe* and *D. discoideum*.

## Concluding remarks

**Fig. 2:**
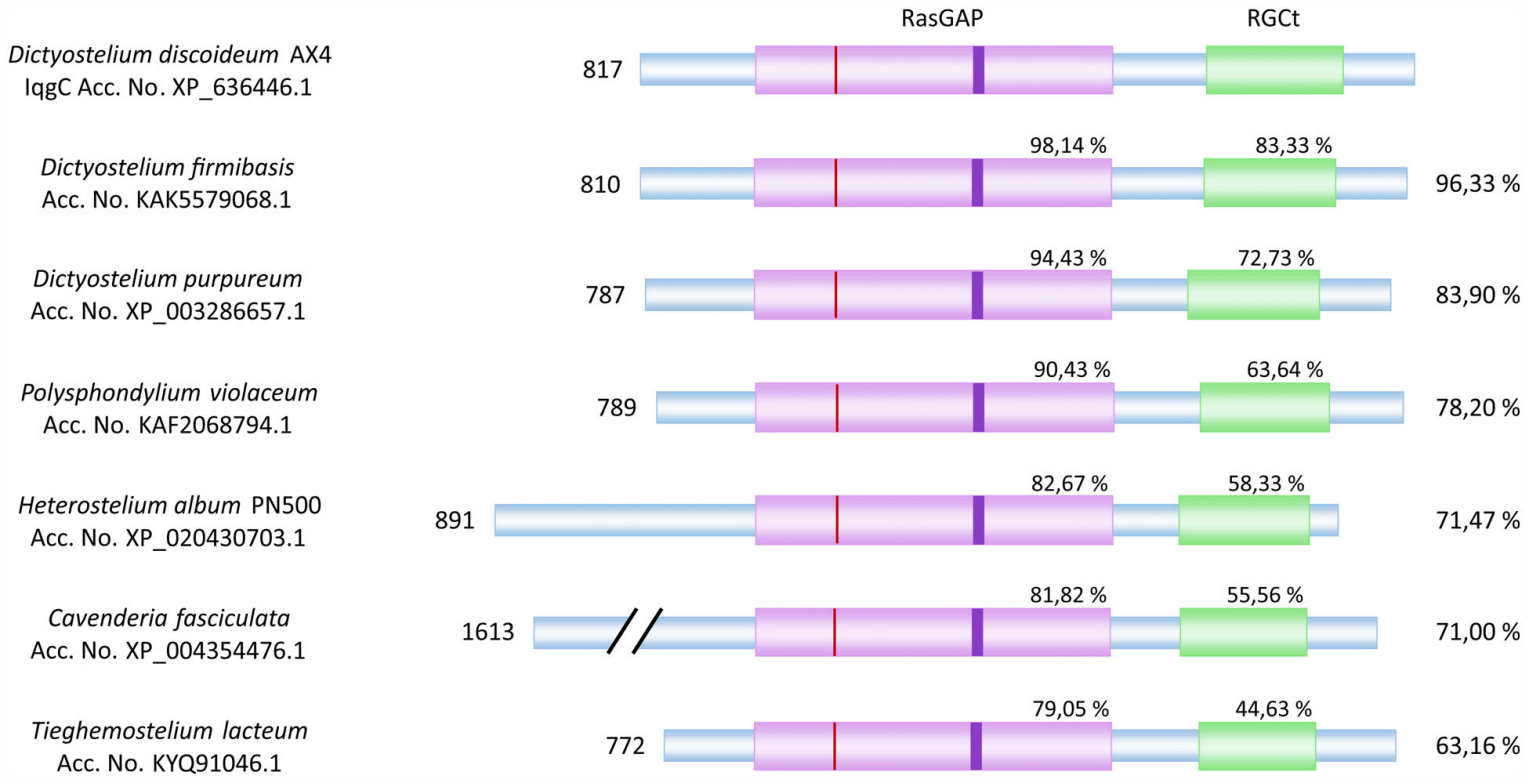
IqgC is conserved in dictyostelids. Schematic representation of the protein domain organisation of IqgC and orthologues from six other dictyostelid species. Protein sequence alignment was performed using NCBI Blastp (https://blast.ncbi.nlm.nih.gov/Blast.cgi). Domain boundaries were determined using InterPro [97]. The orthologue from *C. fasciculata* contains an additional ABC transporter transmembrane domain and was aligned to IqgC starting at the 849th amino acid. The numbers on the left indicate the number of amino acid residues in each protein. The numbers on the right indicate the percentage of total identity compared to IqgC. The numbers above the domains indicate the percentage of identity for each domain compared to the corresponding domain in IqgC. The vertical red bars represent the arginine finger, which is conserved in all proteins. The vertical purple bars represent the FLR motif, which starts as LLR in all proteins, just as in IqgC.

Although it was originally presented as an IQGAP‐related protein [35, 76], phylogenetic analysis suggested that IqgC is more closely related to the GAP1 group of RasGAPs of fungi [10]. A comprehensive characterisation of IqgC performed over the last 7 years shows that its localisation and function are distinct from the truncated IQGAP‐related proteins in *D. discoideum*, thus supporting its reclassification. We therefore suggest that IqgC should be referred to as GAP1‐related RasGAP in the future. In addition to its GAP activity towards RasG, IqgC also binds Rap and Rab GTPases. These interactions play an important role in the regulation of macroendocytosis, cell‐substratum adhesion and directed migration by IqgC. The presence of highly conserved IqgC orthologues in six other dictyostelid species indicates the universal importance of these RGCt‐containing RasGAPs in the physiology of these organisms (Fig. 2).

## Conflict of interest

The authors declare no conflict of interest.

## Author contributions

VF and IW conceptualised the paper; VF, DP, LM, and IW reviewed the literature; VF and IW wrote the paper; DP made the figures. All authors edited and approved the manuscript.
